# Vascular Perfusion Variability in Diabetic Retinopathy: A Sequential Interscan Optical Coherence Tomography Angiography Assessment

**DOI:** 10.3390/jcm14072312

**Published:** 2025-03-28

**Authors:** Giulia Gregori, Lorenzo Mangoni, Alessio Muzi, Veronica Mogetta, Ramkailash Gujar, Mario Chiapponi, Daniela Fruttini, Rosa Dolz-Marco, Jay Chhablani, Cesare Mariotti, Marco Lupidi

**Affiliations:** 1 Eye Clinic, Department of Experimental and Clinical Medicine, Polytechnic University of Marche, 60126 Ancona, Italy; g.gregori98@gmail.com (G.G.);; 2 Humanitas Gradenigo Hospital, 10153 Torino, Italy; 3 Cornea and Stem Cells Department, Dr. Shroff’s Charity Eye Hospital, Daryaganj, New Delhi 110002, India; rkgaiims99@gmail.com; 4 Department of Neurosurgery, Polytechnic University of Marche, 60131 Ancona, Italy; 5 Department of Medicine and Surgery, University of Perugia, 06129 Perugia, Italy; 6 Unit of Macula, Oftalvist Clinic, 46004 Valencia, Spain; 7 Department of Ophthalmology, UPMC Eye Center, University of Pittsburgh, Pittsburgh, PA 15213, USA; 8 Fondazione Italiana Macula ETS, Dipartimento di Neuroscienze, Riabilitazione, Oftalmologia, Genetica e Scienze Materno-Infantili (DINOGMI), University Eye Clinic, 16132 Genova, Italy

**Keywords:** diabetic retinopathy, optical coherence tomography, optical coherence tomography angiography, sequential en-face OCT angiogram

## Abstract

**Background/Objectives**: The aim of this study was to quantify the interscan variability of vascular perfusion in patients with diabetic retinopathy (DR) using sequential optical coherence tomography angiography (OCT-A) scans. **Methods**: Patients with low-grade DR (DRSS score 20) underwent five sequential OCT-A imaging sessions. Vessel perfusion density (VPD) values of the superficial capillary plexus (SCP), intermediate capillary plexus (ICP), deep capillary plexus (DCP), and choriocapillaris (CC) were computed using ImageJ Version 1.54 software via a binarization process. The binarized images were analyzed to compute VPD using vessel density plugins. Inter-session variability was assessed using the coefficient of variation (CV) and intraclass correlation coefficient (ICC). The results were compared with an age-matched group of healthy controls. **Results**: A total of 16 eyes from 16 patients with DR (6 females, 37.5%) and 16 eyes from healthy controls (6 females, 37.5%) were included. Mean VPD values for SCP, ICP, DCP, and CC in patients with DR were 23.64, 13.64, 15.31, and 36.57, respectively. Corresponding values for the healthy subjects were 37.65, 23.32, 22.05, and 49.00, respectively. No significant differences were found in VPD across consecutive scanning sessions (*p* > 0.05) in either group. However, significant differences in VPD were observed between patients who were healthy and patients with diabetes. **Conclusions**: Sequential OCT-A imaging demonstrated no significant inter-session variability in both healthy subjects and subjects with diabetes. The Spectralis OCT-A system showed high reliability for imaging both healthy and diseased conditions.

## 1. Introduction

Diabetic retinopathy (DR) is a serious complication of diabetes mellitus and is currently the leading cause of blindness and vision loss in developed countries [1,2,3]. It is characterized by specific retinal damage that plays a major role in the visual morbidity of affected patients. Microvascular alterations have been shown in eyes with diabetes mellitus, including basement membrane thickening, pericytes and smooth muscle cell loss, endothelial apoptosis, and hypoxic inner retina, ultimately leading to late-stage pathologic angiogenesis with abnormal preretinal microvessels [4,5].

Dye-based angiographies, such as fluorescein angiography (FA), indocyanine green angiography (ICGA), and ultrawide-field color fundus imaging, have been widely used and have played an important role in the diagnosis and management of various retinal vascular diseases, including diabetic retinopathy, for many years.

Optical coherence tomography angiography (OCT-A) is a noninvasive imaging technology changing diagnostic approaches for retinal diseases [6]. OCT-A enables the depth-resolved visualization of retinal and choroidal vascularization by assessing the decorrelation of the signal between static and non-static tissue, specifically the perfused vessels, with a resolution close to the histologic representation of the macular circulation. OCT-A allows for the clear visualization of all the vascular components, including arterioles, capillaries, and venules, whereas the visualization of capillary structure and the foveal avascular zone is limited to FA and ICGA [5,7]. OCT-A C-scans (en face) provide three-dimensional images of all vascular layers of the retina, representing a useful tool in the qualitative and quantitative assessment of retinal, choroidal, or optic nerve vessels in diabetic retinopathy. Moreover, it does not require the administration of intravenous dye, reducing the risk of potential adverse events. Additionally, interactive image evaluation can also help identify possible artifacts, ensuring accurate interpretation [8].

Few studies have characterized microvascular changes such as vascular density and the foveal avascular zone (FAZ) in different retinal plexuses of patients with DR using OCT-A [9,10,11,12]. Although many studies have already described the high degree of accuracy and reproducibility of OCTA instruments in healthy subjects, the chance of incurring measurement errors is yet to be defined for sequential scans [13,14,15,16,17,18,19]. Czako C et al. investigated the intrasession and between-visit reproducibility of OCT-A acquisitions in patients with diabetes, highlighting the consistent reproducibility of vessel density values, notably in the parafoveal ring and the macular area, although the variability of inter-session OCT-A measurements of retinal vasculature in DR has yet to be evaluated [20].

In this study, we evaluate the inter-session variability of quantitative variables using sequential en-face OCT angiograms for all retinal capillary plexuses. Therefore, the purpose of our study was to evaluate the variability of quantitative retinal microvasculature parameters using a single Spectralis OCT-A device for sequential scans in patients with low-grade DR.

## 2. Materials and Methods

### 2.1. Study Design

This was an observational, cross-sectional study involving patients affected by DR, conducted at the Eye Clinic, Polytechnic University of Marche, Ancona, Italy. Prior approval was obtained from the Institutional Review Board IEC Marche (nr. 2572/108/24), and the procedures conformed to the tenets of the Declaration of Helsinki. Written informed consent was obtained from all participants prior to enrollment.

### 2.2. Study Population

Between May and August 2024, 16 consecutive patients with DR were enrolled in this study. Inclusion criteria were low-grade DR (DRSS score 20), age older than 18 years, treatment-naïve DR, and no history of other previous or ongoing diseases/medications that could potentially affect retinal or choroidal anatomy/physiology. Exclusion criteria included any history or clinical evidence of other retinal diseases, glaucoma, ocular surgery within the last six months, refractive error ≥ 4 diopters, and medium opacities that could affect the quality of the OCT-A scans (quality index {QI} lower than 30 dB). All participants underwent a comprehensive ocular examination, including best-corrected visual acuity (BCVA), slit-lamp biomicroscopy, intraocular pressure (IOP) measurement, fundus evaluation, and sequential OCT-A imaging (Spectralis HRA + OCT2, Heidelberg Engineering, Germany) between May and August 2024. Hemodynamic parameters were recorded as part of routine systemic evaluations during baseline visits.

During this period, 16 healthy controls were enrolled and underwent identical evaluations as the study patients.

### 2.3. OCT-A Acquisition

Sequential en-face OCT-A images were obtained employing the same OCT-A apparatus (OCT-A, SpectralisHRA-OCT2, Heidelberg Engineering, Heidelberg, Germany) that was capable of acquiring 85,000 A-scans per second with a 3.9 µm axial and 6 µm lateral resolution. This device used a light source of 870 nm with a bandwidth of 50 nm, and the built-in TruTrack Active Eye Tracking software was applied to reduce motion artifacts.

The acquisitions were performed applying the high-resolution pre-set, with 10° × 10° (3 mm × 3 mm), 15° angle volume scans (512 B-scans with 6 µm of spacing between consecutive B-scans, utilizing an averaging process of 5 frames/scan) centered on the foveal area. For each eye, 5 sequential OCTA scans were obtained by a single experienced operator (R.G.) focusing on the same macular area. There was no resting time interval between the two scans, and no artificial tears were used between scans. The viewer software (Heyex Software, version 1.9.201.0, Heidelberg Engineering, Heidelberg, Germany) was used to assess C-scan (i.e., “en-face”) angiograms. The automated segmentation algorithm of the Heyex software was used to assess the superficial capillary plexus (SCP), intermediate capillary plexus (ICP), deep capillary plexus (DCP), and choriocapillaris (CC). The boundaries set to acquire the SCP capillaries consisted of the internal limiting membrane (ILM) and the posterior boundary of the inner plexiform layer (IPL), including the nerve fiber layer and ganglion cell layer. For the ICP, the posterior boundary of the IPL and the inner boundary of the inner nuclear layer (INL) were considered. The DCP capillaries were isolated between the inner boundary of the INL and the posterior boundary of the outer plexiform layer (OPL). Finally, the CC capillaries were outlined in a 30 µm thick layer posterior to the retinal pigment epithelium–Bruch’s membrane junction. The projection artifact removal (PAR) tool was used to remove artifacts from the OCT-A images, and a contrast setting of 1.4 was applied to all images during segmentation.

### 2.4. Quantitative Assessment of OCT-A Images

In the current study, vessel perfusion density (VPD) was measured in all C-scan angiograms using ImageJ software version 1.48 (National Institute of Health, Bethesda, MD, USA). For the assessment of VPD, all 5 en-face images were stacked and then aligned using the *stacker* plugin in ImageJ software before proceeding to their binarization. The binarization of stacked images was performed using the binary tool in ImageJ with the default method and background light settings. After binarization, the stacked images were separated into 5 separate images. Then, the VPD (defined as the ratio of the area occupied by vessels with flowing blood divided and the total measurement area) was calculated. This procedure was followed for all the en-face OCT-A images of the SCP, ICP, DCP, and CC for both the DR and control cohorts.

### 2.5. Statistical Analysis

The obtained VPD values were assessed using the Shapiro–Wilk test in order to determine if the data were distributed normally. Based on the results obtained, the Friedman test was used to calculate the mean value, standard deviation (SD), and coefficient of variation (CV) between the 5 en-face OCT-A for each eye. The resulting CV values were, in turn, studied to obtain the mean values, SD, and CV in the SCP, ICP, DCP, and CC layers between all eyes of each cohort. The quantitative data were subjected to statistical analysis using the t-test to assess significant differences between groups. In addition, the intraclass correlation coefficient (ICC) was calculated to confirm the reliability of the results in both groups. The interpretation of ICC was based on the following guidelines: below 0.50, “poor”; 0.50–0.75, “moderate”; 0.75–0.90, “good”; >0.90, “excellent” [21]. A *p*-value of <0.05 was considered statistically significant. All statistical analyses were performed using the SPSS Statistical Software v.21 (Statistical Product and Service Solutions Inc., IBM Company, Chicago, IL, USA).

## 3. Results

### 3.1. Demographic Data

A total of 16 eyes of 16 consecutive treatment-naïve patients with low-grade DR (10 males, 62.5%) and 16 eyes of 16 healthy subjects (10 males, 62.5%) were enrolled between May 2024 and August 2024. The mean age was 64.13 ± 10.86 years in the study group and 57.25 ± 14.33 in the control group (*p* > 0.05). In the DR group, the mean duration of diabetes mellitus was 20.06 ± 6.80 years (range 10–38 years), and the mean duration of diabetic retinopathy was 11.63 ± 5.16 years (range 3–18 years). The mean BCVA was 0.04 ± 0.05 logMAR. The mean central macular thickness (CMT) was 231.82 ± 34.47 μm.

### 3.2. Vascular Perfusion Density (VPD)

**Figure 1:**
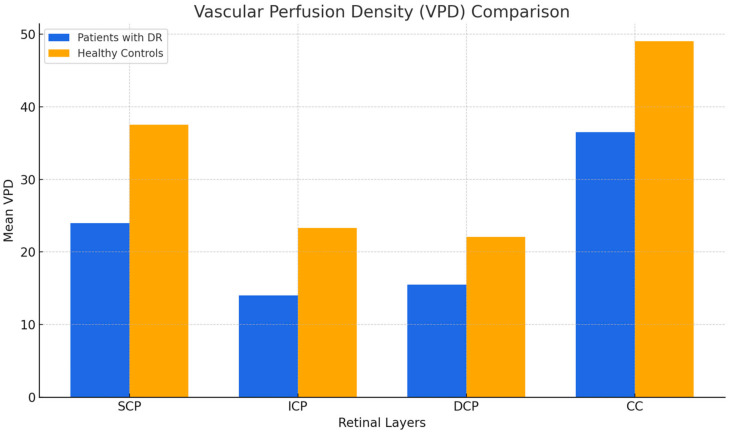
VPD comparison between groups.

In the quantitative assessment, the mean VPD values for the SCP, ICP, DCP, and CC of patients with DR were 23.64, 13.64, 15.31, and 36.57, respectively (as reported in Table 1. The mean VPD for the SCP, ICP, DCP, and CC of the healthy subjects were 37.65, 23.32, 22.05, and 49.00, respectively (as reported in Table 2). No statistically significant differences were reported when comparing the quantitative parameters across different scanning sessions (*p*-values > 0.05) in both healthy patients and patients with DR. A statistically significant difference was observed between healthy patients and those with DR for VPD in each layer analyzed (*p* < 0.001) (Table 3 and Figure 1).

### 3.3. Coefficient of Variation

**Figure 2:**
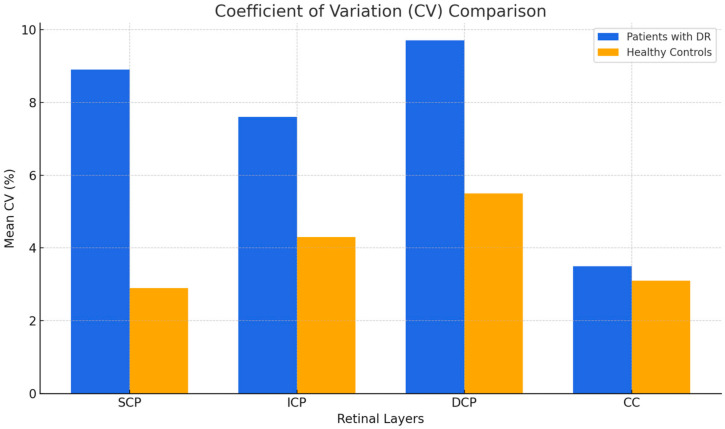
CV comparisons between groups.

The coefficients of variation (CVs) for the SCP, ICP, DCP, and CC of patients with and without DR were also evaluated. The SCP, ICP, DCP, and CC of the patients with DR demonstrated mean CVs of 8.9%, 7.6%, 9.7%, and 3.5%, respectively. The SCP, ICP, DCP, and CC of the healthy patients demonstrated mean CVs of 2.9%, 4.3%, 5.5%, and 3.1%, respectively. The mean CVs in all four layers of patients with DR were higher compared to the corresponding values in healthy patients. Notably, the differences in the CVs of vessel perfusion density registered at the levels of SCP, ICP, and DCP in patients with DR were statistically significant (*p* = 0.0093, *p* = 0.0226, *p* = 0.0434, respectively, as reported in Table 4 and Figure 2).

**Figure 3:**
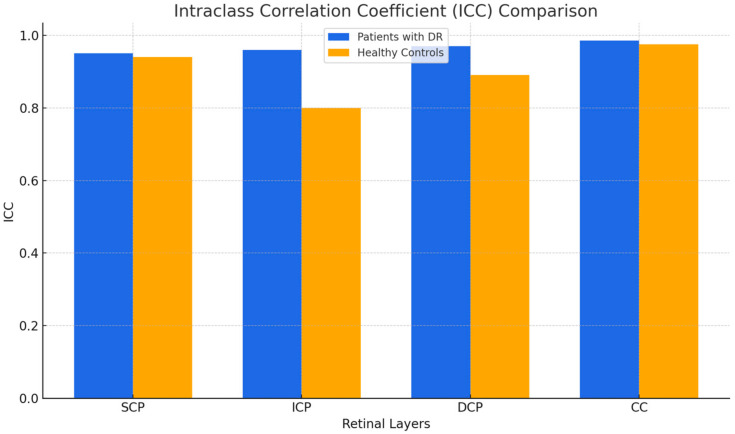
ICC comparisons between groups.

The mean interclass correlation coefficient (ICC) values for the SCP, ICP, DCP, and CC of the control group were 0.944, 0.803, 0.891, and 0.979, respectively. The mean ICC values for the SCP, ICP, DCP, and CC of the patients with DR were 0.954, 0.965, 0.979, and 0.989, respectively. The mean ICC values for both the control group and patients with DR were calculated based on five measurement sessions. Thus, all ICC values ranged between “good” and “excellent” (reported in Table 5 and Figure 3).

## 4. Discussion

En-face OCT angiography provides detailed information about retinal blood flow. However, retinal blood flow can vary between scans, and there is currently a lack of studies evaluating retinal blood flow using sequential en-face OCT angiograms. To our knowledge, this is the first study to quantitatively and qualitatively evaluate the VPD of sequential en-face OCT angiograms in patients with low-grade DR. In the current study, we found that the mean VPD in patients with diabetes was significantly lower compared to healthy subjects for all the plexa analyzed (SCP, ICP, DCP, and CC). Additionally, the CVs of VPD were significantly higher in patients with diabetes compared to healthy subjects in the SCP, ICP, and DCP, indicating more significant variability and abnormalities in capillary blood flow among patients with DR. In line with the findings of previous studies on healthy subjects [13,22], we also found that OCT angiography provides excellent inter-session repeatability of VPD in patients with and without DR. In our study, the CV and ICC values were equal to or higher than the results shown in recent studies, which measured CV and ICC values for VPD intra-visit variability in the SCP [23]. You et al. reported that the intra-visit and inter-visit reproducibility of the VPD of SCP was better in the healthy group than in the group with retinal disease [24]. However, we also found similar CV and ICC values for the ICP, DCP, and CC as the SCP, suggesting good reproducibility. The CVs and ICCs showed better intra-visit repeatability for retinal vessel density in healthy patients using OMAG [17]. Another group also reported high repeatability and reproducibility for the VPD of SCP, showing higher intra-visit CV and ICC values in healthy eyes than in diseased eyes using the same OCTA machine [15]. However, the investigators did not assess retinal measurements for the DCP, ICP, and CC, as automated segmentation can be performed by Heyex software. The low-grade severity of the disease and the absence of macular edema might have contributed to the good repeatability observed in our study subjects. Assessing such variability in advanced disease status and edematous retina would help deepen the understanding of this quantitative parameter.

The high variability among the measurements observed in patients with diabetes reflects the inconsistency of blood flow at the capillary circulation level. Our results are in agreement with those of Yuan et al., who calculated the Pixel Intensity Coefficient of Variation (PICoV) to map the spatial and temporal heterogeneity of perfusion in patients with DR and the control group. Their results showed that PICoV increased based on the severity of DR, indicating more significant variability in retinal capillary flow in these patients. This study highlights how the coefficient of variation can serve as an effective indicator of flow variability in the retinal capillary circulation of subjects with DR. The perfusion heterogeneity variation is more evident in the late stage of DR. This phenomenon could be due to a disruption in the mechanisms regulating capillary flow [25]. The variability of flow at the level of the retinal capillary circulation in subjects with diabetic retinopathy has also been confirmed by fluorescein angiographic techniques combined with adaptive optics scanning light ophthalmoscope (AOSLO) [26].

Uji A et al. demonstrated that en-face averaging significantly improves OCTA image quality and also impacts quantitative measurements [27]. Reduced VPD may lead to ischemia in the fovea, which significantly affects visual acuity in patients with DR [28]. Tang FY et al. found that OCT-A metrics such as the FAZ area, VPD, and VDI were not associated with any systemic (blood sugar, blood pressure, lipids, and body mass index) and demographic (age and duration of diabetes) factors [29]; however, previous studies showed an enlargement of the FAZ in the eyes of patients with diabetes compared to those with healthy eyes on the observation of FA [30,31,32]. In another study, Lei J et al. found that scan order or sequence was also a factor affecting VPD measurements. The primary difference was noticed between the first and last two scans, but no difference was observed between the second and third scans. They also mentioned that signal strength is a significant factor influencing the reproducibility of VPD [15]. Similarly, Lee TH et al. also found that signal strength had a significant negative correlation with the CVs of VPD, indicating higher signal strength, lower CV values, and better reproducibility [23]. In our study, we did not observe any significant difference in the VPD between the first and last scans despite patient fatigue, which was evident in the scans acquired using Spectralis OCT angiography. This indicates that spectralis OCT angiography is more reliable in obtaining sequential OCT angiograms. AI Sheikh et al. also reported good repeatability of OCTA measurement VPD, showing ICC values for the SCP and DCP in scans of healthy subjects at 0.90 and 0.83, respectively [33]. Lee MW et al. found a higher degree of repeatability than anticipated, with an ICC value of 0.737–0.905 and a CV of 5.60–8.86% in retinal diseases between intrascan sessions. They also observed that the repeatability of VPD was affected by BCVA, signal strength, and CMT, but we did not assess these factors in our study [34].

Limitations of the current study include a relatively small sample size and less variability of disease severity. Moreover, OCTA measurements can be affected by several factors, including systemic factors, image acquisition methods, and image quality. In our study, hemodynamic data were collected from enrolled patients when sequential OCT angiogram acquisition was performed. Another limitation is that we did not include all severities of patients with DR, which might have influenced study results. However, the present study is the first to report good reproducibility of sequential en-face OCT angiograms in patients with low-grade DR for the VPD using a single device, which had not been previously evaluated.

## 5. Conclusions

In conclusion, this study confirms the high degree of repeatability and reliability of OCT angiography in measuring vascular changes in patients with diabetic retinopathy and healthy subjects. The differences in vessel density detected between the two cohorts are therefore attributable to an actual alteration in vascular perfusion and confirm the nature of the vascular changes associated with diabetic retinopathy. Based on the data collected, we can hypothesize that sequential angio-tomographic examinations play a significant role. Larger assessments could guide us toward identifying a specific threshold value in terms of the repeatability of the examination, which could discriminate between an actual perfusion anomaly and an intrinsic technical limit. The high degree of variability among the measurements observed in patients with diabetes reflects the inconsistency of blood flow at the capillary circulation level. This parameter could be adopted in the future as a useful tool to predict the risk of progression to more severe forms of DR. Future studies will be necessary to further assess the variability of these retinal measurements using sequential OCT angiograms in advanced diabetic retinopathy.

## Figures and Tables

**Table 1:** Patients with DR.

	SCP			ICP			DCP			CC		
	Mean	SD	CV	Mean	SD	CV	Mean	SD	CV	Mean	SD	CV
Eye 1	24.40	3.23	13.2%	13.21	1.62	12.3%	12.47	0.84	6.7%	37.30	0.57	1.5%
Eye 2	25.18	0.61	2.4%	13.58	0.79	5.8%	14.91	1.03	6.9%	31.29	1.93	6.2%
Eye 3	29.32	1.56	5.3%	17.39	0.35	2.0%	21.92	0.34	1.5%	38.40	1.05	2.7%
Eye 4	14.96	2.29	15.3%	7.71	1.14	14.7%	9.65	1.97	20.5%	33.63	1.14	3.4%
Eye 5	19.34	2.69	13.9%	10.12	1.27	12.6%	9.27	1.29	14.0%	29.63	3.26	1.0%
Eye 6	18.54	3.62	19.5%	10.88	2.42	22.2%	10.05	2.22	22.1%	30.49	2.85	9.3%
Eye 7	24.39	0.66	2.7%	14.37	0.42	2.9%	18.30	0.15	0.8%	44.15	0.53	1.2%
Eye 8	28.85	2.37	8.2%	13.82	0.93	6.8%	18.77	1.21	6.5%	44.13	0.92	2.1%
Eye 9	23.88	2.70	11.3%	16.89	1.19	7.1%	16.69	1.28	7.7%	34.90	0.76	2.2%
Eye 10	25.79	3.14	12.2%	11.20	1.08	9.6%	7.31	1.81	24.8%	29.37	0.56	1.9%
Eye 11	26.73	2.57	9.6%	15.90	0.85	5.3%	17.43	0.81	4.7%	47.32	1.73	3.7%
Eye 12	31.77	0.62	1.9%	15.35	0.35	2.3%	18.99	0.72	3.8%	45.52	0.35	0.8%
Eye 13	20.19	0.41	2.0%	12.72	0.44	3.5%	13.16	0.45	3.4%	39.96	0.74	1.9%
Eye 14	22.78	0.41	1.8%	13.49	0.49	3.7%	15.91	2.75	17.3%	39.32	0.86	2.2%
Eye 15	16.68	2.73	16.3%	15.21	1.21	8.0%	19.53	2.26	11.6%	30.92	1.15	3.7%
Eye 16	25.37	1.57	6.2%	16.36	0.59	3.6%	20.58	0.76	3.7%	28.80	0.88	3.0%
Mean value	23.64			13.64			15.31			36.57		

SCP, superficial capillary plexus; ICP, intermediate capillary plexus; DCP, deep capillary plexus; CC, choriocapillaris.

**Table 2:** Control group.

	SCP			ICP			DCP			CC		
	Mean	SD	CV	Mean	SD	CV	Mean	SD	CV	Mean	SD	CV
Eye 1	36.03	1.32	3.7%	24.70	1.17	4.7%	25.14	1.36	5.4%	59.16	1.27	2.1%
Eye 2	37.01	1.37	3.7%	23.86	1.50	6.3%	23.21	1.36	5.8%	51.41	1.16	2.2%
Eye 3	39.52	1.29	3.3%	24.87	0.34	1.4%	24.01	1.12	4.7%	51.37	0.48	0.9%
Eye 4	35.97	1.33	3.7%	21.75	0.70	3.2%	18.41	1.59	8.6%	46.91	3.55	7.6%
Eye 5	38.19	1.71	4.5%	22.69	0.67	3.0%	19.31	0.47	2.4%	46.48	2.17	4.7%
Eye 6	41.30	0.29	0.7%	24.46	0.29	1.2%	23.13	0.44	1.9%	55.51	0.91	1.6%
Eye 7	40.70	0.77	1.9%	24.08	0.33	1.4%	23.28	0.56	2.4%	55.51	0.97	1.8%
Eye 8	34.44	0.85	2.5%	23.13	0.20	0.9%	23.12	0.48	2.1%	44.18	0.98	2.2%
Eye 9	34.90	0.46	1.3%	23.20	0.45	1.9%	21.77	0.51	2.3%	43.99	1.10	2.5%
Eye 10	36.49	1.01	2.8%	23.62	0.73	3.1%	20.45	0.72	3.5%	47.99	0.90	1.9%
Eye 11	37.55	1.62	4.3%	24.86	0.50	2.0%	21.84	0.55	2.5%	48.63	0.61	1.3%
Eye 12	36.78	2.37	6.5%	20.82	2.45	11.8%	21.24	2.36	11.1%	38.44	2.05	5.3%
Eye 13	41.00	1.09	2.7%	23.67	0.96	4.1%	26.72	4.74	17.7%	49.27	1.45	2.9%
Eye 14	36.73	1.00	2.7%	21.17	3.05	14.4%	19.85	0.57	2.9%	44.17	1.75	4.0%
Eye 15	37.39	0.83	2.2%	23.76	1.24	5.2%	21.52	2.73	12.7%	51.69	3.02	5.8%
Eye 16	38.42	0.22	0.6%	22.42	0.93	4.2%	19.81	0.47	2.4%	49.29	1.20	2.4%
Mean value	37.65			23.32			22.05			49.00		

SCP, superficial capillary plexus; ICP, intermediate capillary plexus; DCP, deep capillary plexus; CC, choriocapillaris.

**Table 3:** VPD comparison.

	*p*	Diabetic Patients		Control Patients	
		Mean	SD	Mean	SD
SCP	<0.001 *	23.64	4.67	37.65	2.08
ICP	<0.001 *	13.64	2.65	23.32	1.26
DCP	<0.001 *	15.32	4.50	22.05	2.23
CC	<0.001 *	36.57	6.33	49.00	5.18

* Significant at the 0.05 probability level.

**Table 4:** Coefficients of variation.

	*p*	Diabetic Patients		Control Patients	
		Mean	SD	Mean	SD
SCP	0.0093 *	8.9%	0.06	2.9%	0.01
ICP	0.0226 *	7.6%	0.06	4.3%	0.04
DCP	0.0434 *	9.7%	0.08	5.5%	0.05
CC	0.8653	3.5%	0.03	3.1%	0.02

* Significant at the 0.05 probability level.

**Table 5:** Intraclass correlation coefficients.

	Diabetic Patients			Control Patients		
	ICC_1_	l.r.l._2_	u.r.l._3_	ICC	l.r.l.	u.r.l
SCP	0.954	0.905	0.982	0.944	0.884	0.978
ICP	0.965	0.928	0.986	0.803	0.594	0.922
DCP	0.979	0.956	0.992	0.891	0.775	0.957
CC	0.989	0.977	0.996	0.979	0.957	0.992

ICC_1_, intraclass correlation coefficient; l.r.l._2_, lower reference limit; u.r.l._3_, upper reference limit.

## Data Availability

Data are fully available upon specific and motivated requests to the authors.
